# Development, Evaluation, and impLemenTation for guideline adaptation: a quality improvement protocol for the DELTA study in global health practice

**DOI:** 10.1186/s12961-023-01060-z

**Published:** 2023-11-01

**Authors:** Shu Wang, Yuan Zhang, Zhixuan Wen, Yueming Yang, Yuxuan Zhang, Yixiong Geng, Yali Liu, Jianguo Zhang

**Affiliations:** 1 Department of Neurosurgery, Beijing Neurosurgical Institute, Beijing Tiantan Hospital, Capital Medical University, Beijing, 100070 China; 2 Neonatal Center, Beijing Children’s Hospital, Capital Medical University, National Center for Children’s Health, Beijing, 100045 China; 3 Department of Neurology, Beijing Tiantan Hospital, Capital Medical University, Beijing, 100070 China; 4 Department of Hematology, Dalian Municipal Woman and Children’s Medical Center (Group), Dalian, 116000 China; 5 Beijing Children’s Hospital, Capital Medical University, National Center for Children’s Health, Beijing, 100045 China; 6 Center for Clinical Epidemiology and Evidence-Based Medicine, Beijing Children’s Hospital, Capital Medical University, National Center for Children’s Health, Beijing, 100045 China

**Keywords:** Clinical practice guidelines, Adaptation, Global health, Health equity, Quality improvement, Protocol

## Abstract

**Background:**

Guideline adaptation is an emerging field to provide more appropriate recommendations for local clinical practice quality and to promote global health equity. However, its utilization status, adaptation procedures, and related materials remain to be studied.

**Methods:**

This study developed a quality improvement protocol for a study as the Development, Evaluation, and impLemenTation for guideline Adaptation (DELTA) study. Current adapted clinical practice guidelines (CPGs) will be systematically searched. Their characteristics, utilization status, and adaptation procedures will be extracted, compared, and analyzed. Whether these adapted CPGs rigorously followed the instruments and steps of adaptation frameworks will also be appraised. In addition, the advantages and limitations of current adaptation methods and their suitable application situations will be analyzed. In addition, future perspectives as DELTA series and DELTA system, aiming for comprehensively evaluating current needs for guideline adaptation and developing a unified framework and related materials were proposed to improve the acceptability, applicability, and implementation of guideline adaptation in clinical practice. The DELTA series are divided into four phases: phase I in analyzing status, characteristics, and procedures and completeness of adapted CPGs; phase II in analyzing differences, heterogeneity, and implementation between adapted and original CPGs; and phase III in collecting, analyzing, and comparing all available adaptation materials. With these research bases, an international working group will be established in phase IV and will develop unified guideline adaptation materials after Delphi consensus, including adaptation frameworks, appraisal tools and checklists, registries, and databases.

**Discussion:**

Guideline adaptation has been advanced as an efficient way to guide local clinical practice. However, it still faces several major challenges. The proposed DELTA study, series, and system will further contribute to this emerging topic.

*Trial registration*: This study has been registered by the PROSPERO international database. https://www.crd.york.ac.uk/prospero/display_record.php?RecordID=400170.

**Supplementary Information:**

The online version contains supplementary material available at 10.1186/s12961-023-01060-z.

## Introduction

Clinical practice guidelines (CPGs) provide important essential evidence-based clinical recommendations [1]. High-quality and rigorously developed CPGs help health providers perform appropriate clinical practices to improve clinical outcomes, which is also helpful for policy makers and educators and ultimately benefits patients [2, 3]. A large number of CPGs on various topics have emerged globally with the spread of guideline development methods [4]. However, studies have shown that the quality of guideline development, the strength of recommendations, and the applicability of local implementation are uneven [5]. Only limited CPGs were recommended with high quality in methodology and reporting [6–8]. To establish a trustworthy high-quality CPG, a rigorous, complete, evidence-based development procedure with a systematic review of the literature, appraisal of the quality and strength of evidence and recommendations, and consideration of patient and public involvement should be conducted by a multidisciplinary panel of experts [4, 9].

Nevertheless, it might be a challenge to form such a comprehensive expert group and conduct this full CPG development and review procedure for resource-limited (low- and middle-income) countries/regions [6, 8]. Transforming evidence into a clinical decision requires consideration of local situations, which makes direct utilization of existing CPGs developed under different conditions and organizations not advisable [10, 11]. Guideline adaptation from existing CPGs is a sensible cost-effective and less resource-intensive alternative to endorse or modify recommendations for local setting clinical practice, which is an emerging topic attracting scientific interest [12, 13]. In addition, guideline adaptation based on high-quality rigorously developed CPGs promotes global health equity through implementation and adaptation of clinical practice with equivalent methodological rigor and adequate applicability and contextualization for countries/regions with limited resources [11, 14, 15]. Currently, two of the most widely known and used for guideline adaptation [16, 17] are the ADAPTE framework [18] and the Grading of Recommendations Assessment, Development and Evaluation-adoption, adaptation, and de novo development of recommendations (GRADE-ADOLOPMENT) framework [19], while other adaptation methods such as the Making GRADE the Irresistible Choice (MAGIC) [20], the CHOICE-D codesign process [21], and the Guideline Adaptation and Implementation Planning Resource (CAN-IMPLEMENT) [22, 23] have also been proposed. The ADAPTE framework was developed by the ADAPTE group and the Practice Guideline Evaluation and Adaptation Cycle group in 2005 [24] and updated in 2009 [18] through workshops, meetings, specialist consultations, and literature evaluations. It has a detailed resource toolkit and designs a 24-step, 3-phase (set up, adaptation, and finalization) procedure with completed modules and specific tasks [18]. The GRADE-ADOLOPMENT framework was developed by the GRADE working group in 2006 [11] to apply the GRADE approach and its Evidence-to-decision (EtD) framework and tables to guideline adaptation comprising 8 main steps in 3 phases: “identify and prioritize credible existing guidelines or evidence syntheses of interest and relevance”, “evaluate and complete GRADE EtD framework for each recommendation”, and “GRADE-ADOLOPMENT of recommendations in a guideline (adoption, adaptation, and de novo)” [11, 19].

However, the complexity and inconsistency of current adaptation procedures and the lack of a unified adaptation framework, without adequate appraisal tools, and unavailable registries and databases make the development and implementation of guideline adaptation still inadequate and limited [25], suggesting that the methodological and reporting quality of some adapted CPGs remains unsatisfactory [17, 26]. Thus, we proposed this quality improvement protocol of the Development, Evaluation, and impLemenTation for guideline Adaptation study (the DELTA study) to evaluate current utilization, quality, and methods of guideline adaptation and further discussed its future research perspectives (the DELTA series) as well as the preparation for a more scientific and standardized theoretical and practical framework and related materials (the DELTA system). This study series will contribute to ensuring that knowledge from available research is effectively and contextually used to improve global health and health equity, especially in low- and middle-income countries/regions, which can be beneficial for health practitioners, decision makers, educators, and ultimately, for global clinical practice [27].

## Methods

### Study design

This study protocol is proposed for the DELTA study as the first phrase (phrase I) of a study series (the DELTA series). The DELTA study is a retrospective study to evaluate the development and utilization status, methodological quality, and implementation of current adapted CPGs using the two most widely used ADAPTE [18] framework and GRADE-ADOLOPMENT [19] framework, while their original CPGs were also identified for analysis and comparison. Methodological quality will be appraised by assessing whether these adapted CPGs rigorously follow the instruments and steps of adaptation frameworks. In addition, by comparing the adaptation procedures and steps and framework implementations in global clinical practice of these two guideline adaptation frameworks, we will analyze their consistency and heterogeneity, discuss their rigor, efficiency, and transparency and summarize their advantages and limitations in exploring their potential suitable application situations and future improvements. Finally, this study will propose relevant proposals and strategies for the quality improvement of guideline adaptation research.

To support a rigorous and comprehensive study procedure and quality control, the original study will apply systematic search, selection, and data extraction methods according to the requirements of systematic review following relevant items in the PRISMA-P checklist (Additional file 1: Table S1) [28]. Specialists, including clinicians in different fields, methodologists, and researchers with experience in developing and adapting guidelines, have established a research team for this study with an external review team.

In addition, perspectives on future collaboration and study series concerning guideline adaptation as the DELTA series (consisting of phases I–IV) and the preparation for a novel unified guideline adaptation framework and related materials (the DELTA system) are also proposed as future research directions. These studies and frameworks will have their own detailed designs and protocols through a multistage step-by-step development process.

### Eligibility criteria

The inclusion criteria of the DELTA study will be as follows: all adapted CPGs should be either clinical practice guidelines, clinical treatment guidelines, or clinical recommendations with the definition of guideline “adaptation” as “systematic approach for considering the endorsement or modification of guidelines produced in one setting for application and implementation in another as an alternative to de novo guideline development or as a first step in the process of implementation, while preserving evidence-based principles” by Fervers et al. [12, 13, 16]; the adaptation methods used should be either ADAPTE, GRADE-ADOLOPMENT, or combined use of these two methods; publication dates for included studies should be from the initial indexing time of the databases to December 31st, 2021; and all included CPGs should be in English to represent internationally recognized guidelines. Documents that are not adapted CPGs, which include updated guidelines, translated guidelines, interpreted guidelines and brief versions, will be excluded. In addition, duplicate published adapted CPGs were also excluded.

### Search strategy

The following databases and search engines will be systematically searched for potential adapted CPGs: the medicine database as PubMed/MEDLINE (pubmed.gov), Web of Science (webofknowledge.com), and Scopus (scopus.com); CPG databases as Guidelines International Network (GIN; g-i-n.net); Agency for Healthcare Research and Quality/National Guidelines Clearinghouse (AHRQ/NGC; ahrq.gov), and National Institute for Health and Care Excellence (NICE; nice.org.uk); databases of specialized CPG development organizations as Scottish Intercollegiate Guidelines Network (SIGN; sign.ac.uk), Canadian Medical Association (CMA; joulecma.ca) and New Zealand Guidelines Group (NZGG; moh.govt.nz); the CPG registry as Practice guideline REgistration for transPAREnc (PREPARE; guidelines-registry.cn); and the supplementary search engine as Google Scholar (scholar.google.com). In addition, the original CPGs of all included adapted CPGs were also searched and identified. The language limit was set as “English”, and the publication time limit was “from the initial indexing time of the databases to December 31st, 2022”. The search terms will be designed with a combination of free words and MeSH terms (if available). Key search terms will include guideline restriction, “Practice Guideline (Publication Type)” or “Guideline* (* for wildcard)” or “Guidelines as topic (M, for Mesh terms)” or “Guidance*” or “Recommendation*” or “Consensus*”; and topic restriction, “Modify” or “Modifi*” or “Adapt*” or “GRADE-ADOLOPMENT”.

### Selection and data collection

Two researchers will independently conduct the study selection and data collection procedures. After that, they will perform cross-checking and discussions for validation. If disagreements occurred, a senior expert such as Prof. Y.L. made the final decision after reviewing the original records.

**Fig. 1:**
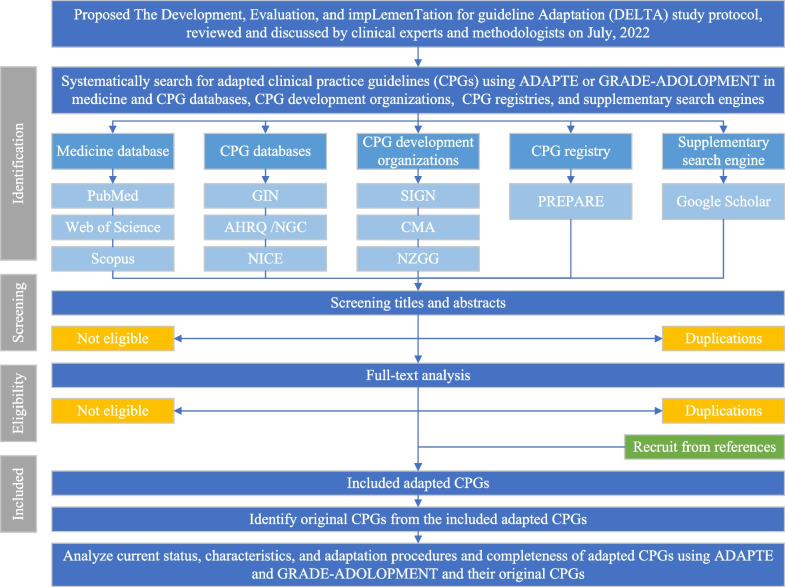
Flow diagram of the systematic search, study selection, data collection, and methodology assessment procedures

The study selection will contain two major steps: screening and eligibility. For the screening step, the researchers will conduct deduplication with assistance from the reference management software (Endnote; Clarivate Analytics, MA, USA, version as used) and will screen titles and abstracts for noneligible studies. Regarding the eligibility step, the researchers will analyze the full texts of studies that pass the screening step for further deduplication and eligibility evaluation. After reviewing the full text, the reviewers will also search for adapted CPGs from references and citations of previously included studies to prevent omissions. These systematic searching and selection procedures will be based on and reported according to the Preferred Reporting Items for Systematic reviews and Meta-Analyses (PRISMA) 2020 statement [29]. After that, original CPGs will be identified for all included adapted CPGs. The study procedures, including systematic search, study selection, data collection, and methodology assessment, are shown in a flow diagram (Fig. 1).

The data collection procedure will be performed to extract data on study characteristics for adapted and original CPGs and record adaptation methods used. Detailed study variables reflecting characteristics are shown below.

### Study variables

The main purpose of this study is to analyze the current status, characteristics, and adaptation procedures of adapted CPGs using ADAPTE and GRADE-ADOLOPMENT. Therefore, we plan to collect the following study variables of the included adapted CPGs and their original CPGs to conduct comparisons for different adaptation methods.

1. Year of publication: We plan to extract the published year of the adapted and original CPGs to analyze trends in the utilization of different adaptation methods and time periods of guideline adaptation from the original CPGs.
2. Countries/regions and organizations to develop CPGs: This variable will be collected to locate countries/regions where the adapted and original CPGs were developed, which will be divided into developing and developed countries/regions according to the World Trade Organization (WTO) list. Organizations of CPGs are the group responsible for guideline development. Individuals, few persons, or small teams are considered not to be under organization or group responsibility.
3. Target fields: We will classify the target fields of the CPGs using the International Classification of Diseases 11th Revision (ICD-11) and extract detailed information on their fields of focus.
4. Target population: Each CPG should report a suitable population to implement, such as newborns, infants, pediatrics, teenagers/adolescents, adults, pregnant women, and elderly people, which will be extracted.
5. Evidence-based methodology and EtD frameworks: The methodology of CPG development (evidence-based or not) will also be extracted and documented. Whether the guideline is evidence-based will be evaluated by assessors after reviewing the full text according to the definition of evidence-based guidelines by the Institute of Medicine of the United States National Academies as “statements that include recommendations intended to optimize patient care and are informed by a systematic review of evidence and an assessment of the benefits and harms of alternative care options” [30]. The other guidelines that lacked evidence-based methods, such as expert opinion-based guidelines, were considered nonevidence-based guidelines. In addition, whether the CPG provides EtD frameworks of the GRADE approach [19, 31, 32] and their methodology in guideline development will also be recorded.

### Adaptation methodology assessments

Two major adaptation methods, including the ADAPTE framework [18] developed by the ADAPTE Collaborative Group and the GRADE-ADOLOPMENT framework [19] developed by the GRADE Working Group, will be included and appraised for their consistency and heterogeneity in comparing their rigor, efficiency, and transparency and exploring their suitable application situations and potential future improvements in the present study. The ADAPTE framework consists of three main phases (set-up, adaptation, and finalization), each with a set of tasks and modules with a total of 24 steps [18] (refer to Table 1 for full steps). The GRADE-ADOLOPMENT framework is a three-phase 8-step process (Groups and roles; Selection of guideline topics; Prioritizing questions for selected guidelines; Using the GRADE Evidence to Decision frameworks; Updating systematic reviews of health effects and identifying local data; Preparing GRADE evidence tables and Evidence to Decision frameworks; Formulating and rating strength of recommendations; Arriving at a final framework for GRADE-ADOLOPMENT) for adoption, adaptation, and de novo development of trustworthy recommendations, including direct adoption of original recommendations, modification of original recommendations and development of new recommendations [19] (Table 1). In addition, the assessment team will also compare the adaptation procedures and content of these adapted CPGs with the steps of adaptation methods to appraise whether these adapted CPGs rigorously followed the instruments, requirements, and steps of adaptation frameworks.

**Table 1:** The adaptation procedures of the ADAPTE framework and the Grading of Recommendations Assessment, Development and Evaluation-adoption, adaptation, and de novo development of recommendations (GRADE-ADOLOPMENT) framework

Phases	Tasks	Associated modules	Steps
The ADAPTE framework			
Phase I—Set up	Prepare for ADAPTE process	Preparation	Step 1. Establish an organizing committee
			Step 2. Select a guideline topic
			Step 3. Check whether adaptation is feasible
			Step 4. Identify necessary resources and skills
			Step 5. Complete tasks for the set-up phase
			Step 6. Write adaptation plan
Phase II—Adaptation	Define health questions	Scope and purpose	Step 7. Determine the health questions
	Search and screen guidelines	Search and screen	Step 8. Search for guidelines and other relevant documents
			Step 9. Screen retrieved guidelines
			Step 10. Reduce a large number of retrieved guidelines
	Assess guidelines	Assessment	Step 11. Assess guideline quality
			Step 12. Assess guideline currency
			Step 13. Assess guideline content
			Step 14. Assess guideline consistency
			Step 15. Assess acceptability and applicability of the recommendations
	Decide and select	Decision and selection	Step 16. Review assessments
			Step 17. Select between guidelines and recommendations to create an adapted guideline
	Draft guideline report	Customization	Step 18. Prepare draft adapted guideline
Phase III—Finalization	External review	External review	Step 19. External review—target audience of the guideline
			Step 20. Consult with endorsement bodies
			Step 21. Consult with source guideline developers
			Step 22. Acknowledge source documents
	Plan for future review and update	Aftercare planning	Step 23. Plan for aftercare of the adapted guideline
	Procedure final guideline	Final production	Step 24. Produce final guidance document
The GRADE-ADOLOPMENT framework			
Phases			Steps
Phase I—Identify and prioritize credible existing guidelines or evidence syntheses of interest and relevance			Step 1. Groups and roles
			Step 2. Selection of guideline topics
			Step 3. Prioritizing questions for selected guidelines
Phase II—Evaluate and complete GRADE evidence to decision framework for each recommendation			Step 4. Using the GRADE Evidence to Decision frameworks
			Step 5. Updating systematic reviews of health effects and identifying local data
			Step 6. Preparing GRADE evidence tables and Evidence to Decision frameworks
			Step 7. Formulating and rating strength of recommendations
Phase III—GRADE-ADOLOPMENT of recommendations in a guideline (adoption, adaptation, and de novo)			Step 8. Arriving at a final framework for GRADE-ADOLOPMENT

GRADE-ADOLOPMENT, the Grading of Recommendations Assessment, Development and Evaluation-adoption, adaptation, and de novo development of recommendations. Detailed description and related toolkit of the ADAPTE frameworks can be found in the ADAPTE manual, which is available from http://www.g-i-n.net by the ADAPTE Collaboration; and the GRADE-ADOLOPMENT framework is available from J Clin Epidemiol. 2017 Jan;81:101–110. by Schünemann et al.

The present study also applied quality control for adaptation methodology assessments. We plan to recruit clinicians who had multiple fields of clinical experience and evidence-based medicine specialists (especially those who had guideline adaptation experiences) to evaluate the adaptation method of adaptation guidelines, which contributes to making the appraisal more accurate and reliable. Not only will we train the assessment team and conduct multiround test assessments before the formal adaptation methodology assessment procedure, but a senior expert (Prof. Y.L.) will also randomly select 10% of the assessments for reassessments as quality control, which will help to increase the credibility of the assessment and reduce selection bias. A previous study by the research team reported a similar quality control procedure [6].

### Future perspectives-the DELTA series

**Fig. 2:**
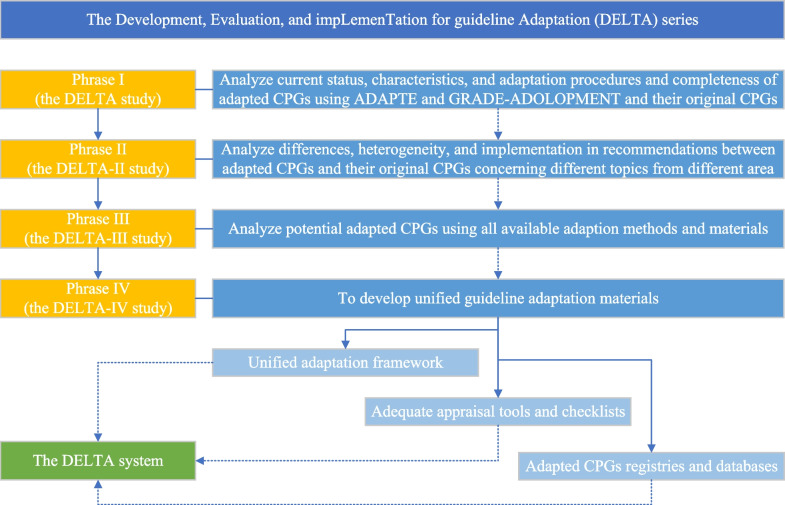
Future perspectives on the Development, Evaluation, and impLemenTation for guideline Adaptation (DELTA) collaboration and materials (the DELTA series and the DELTA system)

Perspectives on collaboration and materials for guideline adaptation are also proposed, which can be future research directions for guideline adaptation. As shown in Table 2, appraisal tools (AGREE II, The Appraisal of Guidelines for Research & Evaluation Instrument [33–35]; AGREE-REX, AGREE- Recommendation Excellence [36]; and RIGHT, Reporting Items for practice Guidelines in HealThcare [37] statement), registries (PREPARE, Practice guideline REgistration for transPAREnc), and databases (GIN, Guidelines International Network; AHRQ/NGC, Agency for Healthcare Research and Quality/National Guidelines Clearinghouse; and NICE, National Institute for Health and Care Excellence) have been established for general CPGs, while they are still limited for adapted CPGs. Only an extension checklist of the RIGHT statement for the reporting of adapted CPGs (the RIGHT-Ad@pt Checklist [38]) has been proposed recently. Thus, we designed a four-phase study series (the DELTA series) to provide full insight for guideline adaptation (Fig. 2). The DELTA-II study will be conducted to analyze differences, heterogeneity, and implementation in recommendations between adapted CPGs and their original CPGs, which will cover different topics from different areas. In particular, CPGs concerning Traditional Chinese Medicine (TCM) and other complementary medicine will also be considered for a more complete coverage. The DELTA-III study will collect, analyze, and compare all available adapted CPGs and potential adaptation methods and materials in summarizing detailed procedures and contents in developing adapted CPGs (such as topics, search strategies, databases, evaluation, and decision) and analyzing their strengths and weaknesses to identify research gaps and needs of guideline adaptation. With these research bases, we will establish an international working group to develop a more scientific and standardized theoretical and practical framework and related materials for guideline adaptation materials (the DELTA system) after Delphi consensus [39]. The DELTA system will consist of a unified adaptation framework, adequate appraisal tools and checklists, and adapted CPG registries and databases, which will be the main direction of the DELTA-IV study as the ultimate goal. The developed adaptation framework will consider the advantages and limitations of current frameworks and provide rigorous, concise, efficient, and transparent procedures and steps. The key instruments will consider the needs of guideline developers and related researchers and other stakeholders with external reviews, which will mainly include how to form a group and choose topics and keywords, what the basic databases and search engines are, how to screen literature and collect information, how to determine key questions and perform quality assessments, how to form CPG elements through literature contents and local situations, how to involve the public and different fields, and other requirements in quality control, conflict of interest, reporting checklists, and updates. In addition, different areas (such as TCM [40]) may provide extensions to meet the requirements of different fields and improve the universality of the DELTA system.

**Table 2:** Appraisal tools, registries, and databases of general and adapted guidelines

Subject	General guidelines	Adapted guidelines
Appraisal tools	AGREE II (and Reporting Checklist)	RIGHT-Ad@pt Checklist
	AGREE-REX (for recommendations)	
	RIGHT statement	
Registries	PREPARE	N/A
Databases	GIN	N/A
	AHRQ/NGC (the U.S.)	
	NICE (the U.K.)	

AGREE II, The Appraisal of Guidelines for Research and Evaluation Instrument; AGREE-REX, AGREE-Recommendation Excellence; RIGHT, Reporting Items for practice Guidelines in HealThcare; PREPARE, Practice guideline REgistration for transPAREnc; GIN, Guidelines International Network; AHRQ/NGC, Agency for Healthcare Research and Quality/National Guidelines Clearinghouse; NICE, National Institute for Health and Care Excellence; RIGHT-Ad@pt Checklist, RIGHT- Adapted Guidelines

### Statistical analysis

In the present study, continuous variables will be reported as the mean (standard deviation, SD) or median (interquartile range, IQR; P_25_–P_75_) with minimum and maximum values. Categorical variables will be reported as numbers (percentages). The Shapiro‒Wilk test or Kolmogorov‒Smirnov test and Levene’s test will be used for analysis of distributions and variance for appropriate statistical reports and tests. Tests for differences among groups included one-way analysis of variance (ANOVA) or Welch's ANOVA (for multiple continuous variables); two independent samples *t* test or Mann‒Whitney *U* test (for two independent continuous variables); two paired samples *t* test or Wilcoxon test (for two paired continuous variables); and Pearson’s *χ*^*2*^ test or Fisher’s exact test (for categorical variables), as appropriate. In addition, to explore publication trends in adapted CPGs, univariate linear regression will be conducted by using the publication year as a continuous independent variable, and the *β*-value (95% confidence interval, CI) and *P *value will be provided as average annual changes and *P *_for trend_ [41]. In the present study, all statistical tests were two-tailed, and a *P *value < 0.05 was considered statistically significant. All statistical analyses will be conducted by SPSS software (IBM Corporation, NY, USA, version as used).

## Discussion

CPGs are increasingly used around the world to promote beneficial recommendations while discouraging ineffective or possibly dangerous interventions by a systematic review of credible evidence [2, 3, 42]. Guideline adaptation refers to accepting or modifying existing guideline recommendations using an evidence-based approach based on the local medical context, which contributes to providing more appropriate recommendations for clinical practice in different local settings [12, 43]. It not only considers local language, culture, economic levels and social environment but also helps to reduce the time and costs consumed to develop CPGs [44–46], which receives growing scientific interest [10, 47]. In addition, guideline development is a complex and resource-intensive procedure that may not be guaranteed in all countries/regions [13]. Much clinical guidance and many effective interventions are not reaching people who need them most, especially in countries/regions with limited resources, because of shortcomings in experienced health providers and fragmented and overburdened health systems [15, 27, 48]. For low- and middle-income countries/regions, guideline adaptation is a sensible approach for saving resources and receiving clinical recommendations of equivalent quality from developed CPGs with adequate local applicability [6, 8]. Thus, it can be helpful to ensure knowledge and experience spread across barriers from different socioeconomic and healthcare gaps and improve global health and health equity [13, 49, 50].

Currently, two of the most widely known and used for guideline adaptation [16, 17] are the ADAPTE framework [18] and the GRADE-ADOLOPMENT framework [19]. The ADAPTE framework was developed by the ADAPTE Working Group [18], which aimed to provide a systematic approach to adapt guidelines to meet the needs of users with different cultural and organizational contexts [51]. The GRADE-ADOLOPMENT framework was developed by the GRADE Working Group [19] based on their international standardized guideline development procedure. This framework provides a structured approach that combines the adoption, adaptation and de novo development of trustworthy guideline recommendations [52]. The application of the GRADE EtD frameworks makes the adaptation process more transparent and convenient [19]. After more than ten years of development of guideline adaptation, little is known about the development, utilization, and implementation status of guideline adaptation. The DELTA study will search and summarize these questions.

The ADAPTE and GRADE-ADOLOPMENT methods have been widely applied in guideline adaptation. In 2020, Hurtado et al. [53] developed an adaptation guideline for generalized anxiety disorder care in Spain using the ADAPTE method. Kahale et al. [54] applied the GRADE-ADOLOPMENT method to develop an adapted CPG for breast cancer screening. Our research team also had experience developing adapted CPGs for the diagnosis and treatment of primary immune thrombocytopenia in Chinese children by applying the ADAPTE methodology [50]. The ADAPTE method requires the participation of clinical experts, evidence-based medicine experts and patient involvement. This method emerged earlier in 2005 and was updated as the second version in 2009, which is now more widely known and applied [16]. However, this method has plenty of steps to finish an adapted CPG. Studies have shown that adapting a guideline by the ADAPTE method might not require less time than producing a new guideline and is constrained by time and financial resources [49]. The GRADE-ADOLOPMENT method uses the EtD framework to demonstrate the process from extracting evidence-based medical evidence to forming clinical recommendations, which was first introduced in 2007 [19]. This adaptation method makes the adaptation process transparent, which contributes to the credibility and acceptability of the adaptation guidelines [19]. This method transformed from the widely disseminated and used GRADE approach, which suggests a systematic framework in summarizing evidence profiles into succinct, transparent, and informative findings tables to generate recommendations or decisions, demonstrating wide acceptance and credibility with potential impact on health equity [31, 55]. The GRADE-ADOLOPMENT method also has limitations, such as its unstandardized preparation and finalization steps and the EtD frameworks cannot be integrated for all types of studies, which may result in the inability to integrate the evidence for all the literature on the research question [32, 56]. In summary, each of these two adaptation frameworks has its own advantages and shortcomings and may be applicable for different situations, resulting in their inappropriate implementation [17, 26]. Thus, the DELTA study will compare adaptation procedures and steps and framework implementations in global clinical practice of these two guideline adaptation frameworks to analyze their consistency and heterogeneity, discuss their rigor, efficiency, and transparency and summarize their advantages and limitations in exploring their potential suitable application situations and future improvements, which is a major purpose of this study. In addition, some previous studies raised concerns about the methodological and reporting quality of adapted CPGs [17, 26]. Even though some adapted CPGs claimed they applied adaptation methods, whether they rigorously followed the adaptation instrument and steps remains unknown. Thus, it is necessary to further appraise the methodological quality of current adapted CPGs and discuss potential reasons for their incompleteness, which is another major aim of this study. Finally, this study will propose relevant proposal and strategies in quality improvement regarding future guideline adaptation research and enhancement in its implementation.

In addition, the research team conducted a preliminary search using the strategy mentioned above to summarize current guideline adaptation methods and identified 10 major frameworks that have been developed and applied in published adapted CPGs [16, 17], including the ADAPTE framework [18], the GRADE-ADOLOPMENT framework [19], the MAGIC [20], the CHOICE-D process [21], the CAN-IMPLEMENT [22, 23], the Adapted ADAPTE [57], the Alberta Ambassador Program [58], the RAPADAPTE [59], the Royal College of Nursing (RCN) [60], and the Systematic Guideline Review Method (SGR) [61]. Their characteristics and major strengths and limitations are summarized in Table 3. As shown, major limitations of current adaptation methods are complex steps and long process times (the ADAPTE and the Adapted ADAPTE), concise but undetailed adaptation steps and requirements as well as unstandardized preparation and finalization (the GRADE-ADOLOPMENT, the MAGIC, the CAN-IMPLEMENT, the RCN, and the SGR), and only design for specific groups or needs (the CHOICE-D, the Alberta Ambassador Program, and the RAPADAPTE) [16]. Additionally, these various methods have inconsistencies in their procedures and contents, and few of them were developed with a complete establishment process with a multidisciplinary team [25]. Thus, we proposed a more scientific and standardized unified theoretical and practical framework with its related materials as the DELTA system (Table 3). This framework will apply a multistage development process with the following principles: (1) identify research gaps and needs by the DELTA series; (2) establish an international working group of different fields; (3) systematic review, (4) reporting quality evaluation, (5) Delphi expert consensus, (6) public involvement, (7) multidisciplinary consultations, (8) evaluations, quality control, external review, (9) extensions, implementation, and updates. Additionally, we considered the advantages and limitations of current adaptation methods and proposed improvements in establishing a rigorous, concise, efficient, and transparent adaptation framework (Table 3). Previous studies have proven that rigorous, complete, and systematic guideline development procedures [37, 55, 62], appropriate appraisal tools [33, 37], and guideline registries and databases [63] contributed to the improvement of developing methodology and reporting quality [64] while reducing duplication, improving collaboration, and increasing transparency [63] for general CPGs. As a novel emerging topic, most of these materials are not available for adapted CPGs. We also propose the future study series as a DELTA series to establish the DELTA system, which includes a unified adaptation framework, adequate appraisal tools and checklists, and adapted CPG registries and databases; the present DELTA study will provide a fundamental but crucial step for these future directions. With these studies, we hope to contribute to the development of guideline adaptation and make it easier for use and implementation in global clinical practice and to promote guideline adaptation as a useful tool to ensure that all countries/regions have the ability to apply high-quality evidence-based clinical practice, resulting in the improvement of global health and health equity [27].

**Table 3:** Characteristics and major strengths and limitations of current available guideline adaptation methods (*n* = 10); and design, procedures, main contents, and improvements (considering current advantages and limitations) of the Development, Evaluation, and impLemenTation for guideline Adaptation (DELTA) system

Adaptation method	Year	Design	Steps	Strengths	Limitations
The ADAPTE [18]	2005/2009^a^	Workshops, consultations, evaluations	24 steps/3 phases	Completed modules with specific tasks	Complex steps and long process time
The GRADE-ADOLOPMENT [19]	2006	Apply GRADE EtDs tables to adaptation	8 steps/3 phases	Apply the GRADE approach	Unstandardized preparation and finalization
The MAGIC [20]	2014	Apply GRADE approach to adaptation	5 steps	Apply the GRADE approach, concise	Undetailed adaptation steps and requirements
The CHOICE-D [21]	2020	Collaborate with patients and families	9 steps	Consider patient and public involvement	Designed for publics, limited profession
The CAN-IMPLEMENT [22]	2013	Revise ADAPTE, enhance implementation	5 steps	Enhance implementation, concise	Undetailed adaptation steps and requirements
The Adapted ADAPTE [57]	2015	Revise ADAPTE	24 steps/3 phases	Completed modules with specific tasks	Complex steps and long process time
The Alberta Ambassador Program [58]	2006	Collaboration, committees, partnerships	11 steps/3 stages	Considering local knowledge	Designed for primary care, limited profession
The RAPADAPTE [59]	2016	From ADAPTE, use synthesized evidence databases	12 steps	Concise, rapid	Not all databases are available globally
The RCN [60]	2000	Author team initiate	5 steps	Concise	Undetailed adaptation steps and requirements
The SGR [61]	2006	Author team initiate	9 steps	Concise	Unstandardized preparation and finalization
The proposed DELTA system					
Design	Multistage: (1) identify research gaps and needs by the DELTA series; (2) establish international working group of different fields; (3) systematic review, (4) reporting quality evaluation, (5) Delphi expert consensus, (6) public involvement, (7) multidisciplinary consultations, (8) evaluations, quality control, external review, (9) extensions, implementation, updates				
Main contents	Consider needs of guideline developers and related researchers and other stakeholders: how to form a group and choose topics and keywords, what are the basic databases and search engines, how to screen literatures and collect information, how to determine key questions and perform quality assessments, how to form guideline elements through literature contents and local situation, how to involve publics and different fields, and other requirements in quality control, conflict of interest, reporting checklist, and updates				
Improvements					
- Reserving current advantages	(1) completed instrument with specific succinct tasks; (2) consider GRADE approach; (3) public involvement; (4) enhance implementation; (5) consider different environment and fields				
- Solving current limitations	(1) unified standardized theoretical and practical framework with related materials; (2) preinvestigation to identify needs of guideline developers and related researchers and other stakeholders; (3) developers from international working group of different fields multidisciplinary consultations; (4) with references from available methods; (5) rigorous, concise, efficient, and transparent procedures and steps; (6) detailed instrument and procedures; (7) consider different environment, fields, and publics; (8) general databases and search engines				

^a^The ADAPTE framework was firstly developed in 2005 and updated in 2009 (Version 2.0).

*DELTA* Development, Evaluation, and impLemenTation for guideline Adaptation, *GRADE-ADOLOPMENT* Grading of Recommendations Assessment, Development and Evaluation-adoption, adaptation, and de novo development of recommendations, *MAGIC* Making GRADE the Irresistible Choice, *CAN-IMPLEMENT* Guideline Adaptation and Implementation Planning Resource, *RCN* the Royal College of Nursing, *SGR* the Systematic Guideline Review Method, *GRADE* Grading of Recommendations Assessment, Development and Evaluation, *EtD* evidence-to-decision

### Limitations

First, only adapted CPGs in English will be included, and further research may have international collaboration to include guidelines in different languages. Second, even though we will conduct a rigorous training, test evaluation and reassessment procedure, the assessment may relate to subjective judgment, which could introduce bias. Finally, there are also many other guideline adaptation methods, such as the MAGIC [20], the CHOICE-D process [21], and the CAN-IMPLEMENT [22, 23], which will be analyzed in future studies as the DELTA series.

## Conclusion

Guideline adaptation is a sensible, pragmatic, cost-effective, and less resource-intensive alternative to endorse or modify recommendations for local setting utilization, which promotes global health equity and has become an emerging topic attracting scientific interest. However, studies on the current status, characteristics, adaptation procedures, and related materials of guideline adaptation are still limited. The DELTA study, series, and system will contribute to this topic for its future development.

## Research ethics

This study was approved by the ethics committee of Beijing Tiantan Hospital, Capital Medical University. This study was conducted following the 1964 Helsinki Declaration and its later amendments.

### Supplementary Information

**Additional file 1: **PRISMA-P 2015 checklist.

## Data Availability

All data generated or analyzed during this study are included in this published article and its Additional files.

## Abbreviations

CPGs: Clinical practice guidelines
GRADE-ADOLOPMENT: The Grading of Recommendations Assessment, Development and Evaluation-Adoption, adaptation, and de novo development of recommendations
DELTA: The Development, Evaluation, and impLemenTation for guideline Adaptation
EtD: Evidence-to-decision
AGREE: The Appraisal of Guidelines for Research and Evaluation Instrument
